# Substance P blocks ovariectomy-induced bone loss by modulating inflammation and potentiating stem cell function

**DOI:** 10.18632/aging.104008

**Published:** 2020-10-27

**Authors:** Jiyuan Piao, Jeong Seop Park, Dae Yeon Hwang, Youngsook Son, Hyun Sook Hong

**Affiliations:** 1 Graduate School of Biotechnology and Department of Genetic Engineering, College of Life Science, Kyung Hee University, Seochun-dong, Kiheung-ku, Yong In, Republic of Korea; 2 Department of Biomedical Science and Technology, Graduate School, Kyung Hee University, 1 Hoegi-dong, Dongdaemun-gu, Seoul, Republic of Korea; 3 Kyung Hee Institute of Regenerative Medicine (KIRM), Medical Science Research Institute, Kyung Hee University Medical Center, Kyungheedae-ro, Dongdaemun-gu, Seoul, Republic of Korea; 4 East-West Medical Research Institute, Kyung Hee University Hospital, Kyungheedae-ro, Dongdaemun-gu, Seoul, Republic of Korea

**Keywords:** substance P, osteoporosis, regulatory T cell, immune suppression

## Abstract

Osteoporosis is an age-related disease caused by imbalanced bone remodeling resulting from excessive bone resorption. Osteoporosis is tightly linked with induction of chronic inflammation, which activates osteoclasts and impairs osteoprogenitor in bone marrow. T helper 17 (Th17) cells have been recently recognized as one of major inducers in the pathophysiology of bone loss by secreting IL-17. Thus, modulation of Th17 development is anticipated to affect the progression of osteoporosis.

Substance P (SP) is reported to provide anti-inflammatory effects by controlling immune cell profile and also, promote restoration of damaged stem cell niches—the bone marrow—by repopulating BMSCs or potentiating its paracrine ability. This study aimed to explore the therapeutic effects of systemically injected SP on ovariectomy (OVX)-induced osteoporosis.

Resultantly, SP injection obviously blocked OVX-induced impairment of bone microarchitecture and reduction of the mineral density. In osteoporotic condition, SP could ameliorate chronic inflammation by promoting Treg cell polarization and inhibiting the development of osteoclastogenic Th17 cells. Moreover, SP could rejuvenate stem cell and enable stem cells to repopulate and differentiate into osteoblast.

Collectively, our study strongly suggests that SP treatment can block osteoporosis and furthermore, SP treatment provides therapeutic effect on chronic disease with inflammation and stem cell dysfunction.

## INTRODUCTION

Osteoporosis is now a common disease owing to the constant rise in the aging population worldwide [1]. Osteoporosis causes bone fracture, disability, and even death [2]. The underlying cause of osteoporosis is lack of estrogen from post-menopause. Estrogen deficiency is associated with an increase in the apoptosis of osteocytes [3]. Although medications are available, their detrimental side-effects upon prolonged use have raised safety concerns and been related to critical diseases, including osteopetrosis, osteonecrosis, and, even cancer [4–6]. Therefore, it is imperative to develop new treatment regimens for osteoporosis.

Under normal physiological conditions, bone remodeling relies on the differentiation and activation of two different cell types with opposite functions, namely, the osteoclasts that differentiate from hematopoietic stem cells (HSCs) and perform bone resorption and the osteoblasts that are of mesenchymal origin and enhance bone formation [7, 8]. Osteoclast-induced bone loss is mediated by diverse soluble factors, including tumor necrosis factor- alpha (TNF-α), interleukin (IL)-1, and IL-7 [9–11]. Bone marrow-derived mesenchymal stromal cells (BMSCs) have the capacity to self-renew and differentiate into various mesodermal cell types, including osteoblasts and adipocytes [12, 13]. The fate of BMSCs is determined by the BM microenvironment comprising specific cytokines, molecules, and cells [14].

Aging-related dysregulation of tissue homeostasis is caused with a chronic low-grade inflammation. Aging-induced inflammation has been recognized as the main cause of bone microarchitecture impairment [15, 16]. Inflammatory conditions can activate osteoclasts to promote bone resorption and induce functional impairment in BMSCs, thereby channeling their biased differentiation via adipogenesis at the expense of osteogenesis [17]. Estrogen deficiency following menopause results in the creation of an inflammatory microenvironment in the BM via reactive oxygen species (ROS) accumulation that negatively affects bone remodeling [18]. Therefore, inflammation modulation may have major implications in the progression of osteoporosis.

Regulatory T (Treg) and T helper 17 (Th17) cells are subsets of CD4^+^ T cells. The balance between these two cell types plays an important role in sustaining immune homeostasis. CD4^+^Foxp3^+^ Treg cells are specialized Th cell subsets that maintain immune tolerance, prevent chronic inflammatory diseases, and halt autoimmune diseases. In contrast, CD4^+^IL-17^+^ Th17 cells can recruit innate immune cells, including neutrophils and monocytes, and induce inflammatory stimuli and exaggerate disease status [19–22]. IL-17, the most important effector cytokine secreted by Th17 cells, may upregulate the expression of pro-inflammatory cytokines and chemokines, while IL-21 is an autocrine regulatory factor of Th17 cells known to induce Th17 cell differentiation and impair Treg cellular functions [23, 24]. Considering the exclusive development of Treg or Th17 cells from naïve T cells, the microenvironment that promotes Th17 generation may block the development of Treg [25]. It has been recently demonstrated that a quantitative or functional imbalance between Treg and Th17 cells plays a key role in the progression of bone-related diseases such as osteoporosis and that a low ratio of Treg/Th17 is the most commonly observed phenomenon in osteoporosis [26–28].

Aside from inflammation modulation, angiogenesis is common in bone remodeling and known to maintain the synthesis of normal osteoblastic bone matrix with adequate oxygen, minerals, and nutrition [29]. Kusumbe et al. discovered a specific blood vessel called as type H vessel in the BM that is characterized with high CD31 and endomucin expression [30]. Type H vessel modulates the growth of the bone vasculature to preserve osteoprogenitor cells. The number of type H vessels may decrease with aging, eventually declining osteoprogenitor count and bone density [31]. Maintenance of type H vessel is supported by angiogenic growth factors such as platelet-derived growth factor-BB (PDGF-BB) and vascular endothelial growth factor (VEGF), which are reported to be downregulated in osteoporosis [32, 33]. Therefore, conservation of angiogenic factors and type H vasculature may mitigate the reduction in bone density.

Substance P (SP) is an endogenous neuropeptide of the tachykinin family comprising 11 amino acid residues. Its pleiotropic effects are mediated upon binding to neurokinin-1 receptor (NK-1R) [34]. Systemic injection of SP is known to exert therapeutic effects against inflammatory diseases through the suppression of inflammatory responses by increasing the number of Treg cells and M2 monocytes in the circulation, upregulating IL-10 and transforming growth factor-beta (TGF-β) expression, and decreasing TNF-α levels [35–37]. SP, a new BMSC mobilizer [38], can not only restore the BMSC pool by repopulating BMSCs [39] but also block their senescence by potentiating their ability to secrete anti-inflammatory cytokines [40, 41]. Moreover, SP protects the vascular endothelium and promotes angiogenesis to inhibit the progression of vascular diseases [42–44]. Interestingly, the expression of SP in BM is severely decreased and that of its receptor NK-1R is enhanced in osteoporosis [45, 46], indicative of its relevance to bone formation.

Given these multiple functions of SP, we hypothesized that SP treatment blocks bone loss by modulating immune responses, restoring injured BMSCs, and promoting angiogenesis. In this study, we explored whether SP can ameliorate osteoporosis at the preclinical level. To treat experimental osteoporosis, rats were subjected to ovariectomy (OVX) surgery and then intravenously injected with SP. The efficacy and mechanism of action of SP under osteoporotic stress were investigated both in vitro and in vivo.

## RESULTS

### OVX induces bone loss and enriches the Th17 cell pool

OVX induces estrogen deficiency and is a common method to generate an osteoporosis animal model. OVX-induced estrogen deficiency is known to cause systemic inflammation and elevate TNF-α and IL-17 levels to activate osteoclasts and inhibit repopulation and survival of osteoblasts. The consequences include bone loss and a high risk of fracture. To examine the mechanism underlying bone loss following OVX, bone density and Treg/Th17 cell pool were monitored for 16 weeks.

**Figure 1:**
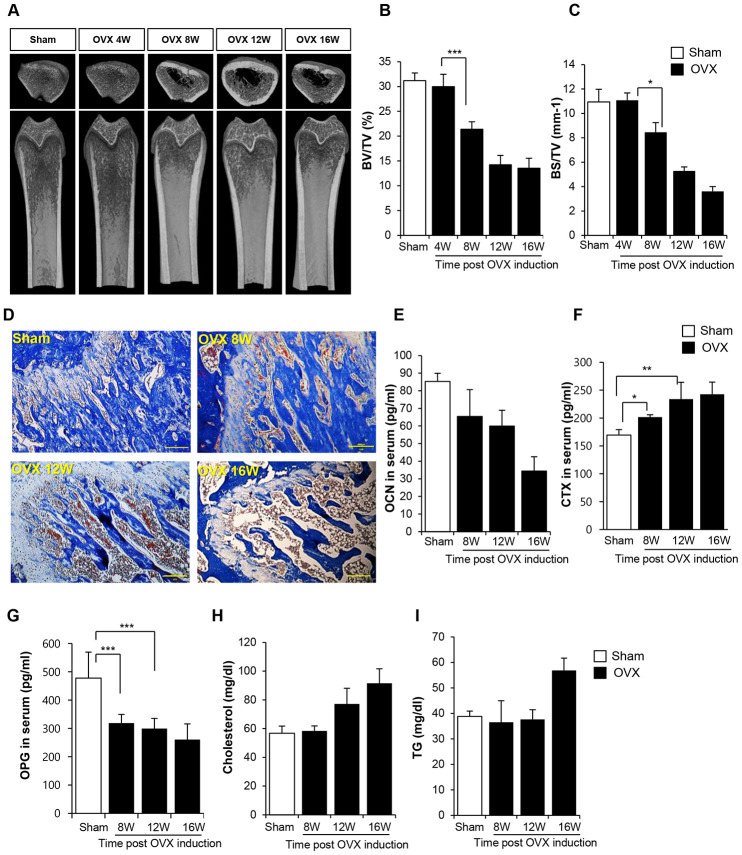
**Ovariectomy (OVX) decreases bone density.** (**A**) Representative femoral microcomputed tomography (μCT) images from OVX rats in different time points. (**B**, **C**) Quantitative analyses of the trabecular bone fraction of femur from rats. (**D**) Masson’s Trichrome staining of distal femoral metaphysis sections from rats after sham-operation and OVX. Scale bar: 200 μm. (**E**–**G**) Biochemical maker for bone loss were examined. OCN, CTX and OPG expression levels in serum were analyzed by ELISA. (**H**–**I**) Cholesterol and TG levels in blood were calculated by ELISA. BV: trabecular bone volume. TV: tissue volume. BS: bone surface. p values of less than 0.05 were considered statistically significant (* p <0.05, ** p <0.01, *** p <0.001). OCN: osteocalcin, CTX: collagen X, OPG: osteoprotegerin, TG: triglyceride. n = 12/group.

Bone loss and inflammation were rarely observed at 4 weeks after surgery in OVX rats (Supplementary Figures 1 and 2). Bone density started to decline from 8 weeks post-surgery, and severe deficiency in bone density was maintained until 16 weeks, as shown in the micro computed tomography (CT) data (Figure 1A–1C). Histological analysis confirmed bone loss and accumulation of the fat (Figure 1D). In comparison with the femur of sham control rats, that of OVX rats showed high levels of the adipose tissue and a low proportion of bone collagen tissue. This observation is indicative of bone loss evident at 8 weeks post-surgery. Analysis of biochemical markers of bone loss showed that the expression of osteocalcin (OCN) and osteoprotegerin (OPG) decreased and that of collagen X (CTX) increased from 8 weeks (Figure 1E, 1F). An increase in cholesterol and triglyceride (TG) levels was observed at 12 and 16 weeks post-OVX, respectively.

Estrogen deficiency promotes Th17-skewed differentiation of naïve T cells and provides IL-17–enriched conditions in peripheral tissue and circulation. Th17 cells are key contributors of bone erosion [47] and directly produce receptor activator of nuclear factor-κB ligand (RANKL) to increase osteoclast formation [48, 49]. IL-17 also enhances the expression of RANKL in osteoblasts [50, 51] and mediates osteoclast-induced secretion of TNF-α and IL-6 in the bone tissue. Therefore, monitoring IL-17 and Th17 cells during disease progression may be important to confirm bone loss.

**Figure 2:**
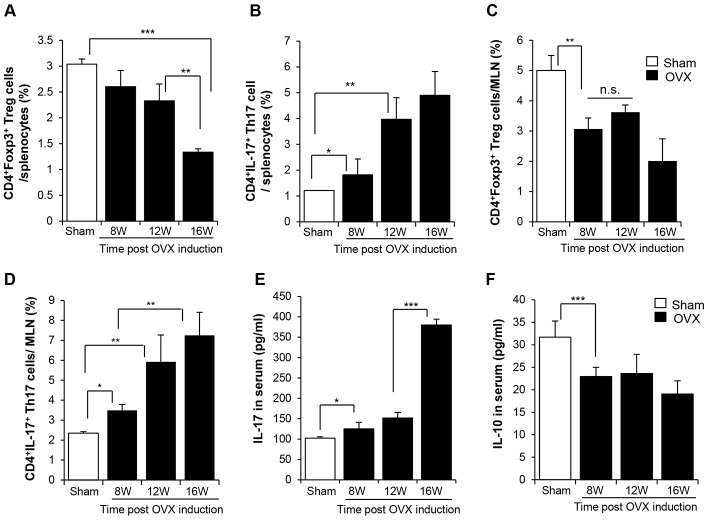
**OVX-induced bone loss enriches of Th17, decreasing Treg in lymphoid organ.** The pool of (**A**, **C**) CD4^+^Foxp3^+^ Treg cells and (**B**, **D**) CD4^+^IL17^+^ Th17 cells in splenocytes and MLN were analyzed by FACS. (**E**, **F**) IL-17 and IL-10 in serum were quantitatively analyzed by ELISA. p values of less than 0.05 were considered statistically significant (* p <0.05, ** p <0.01, *** p <0.001). MLN: mesenteric lymph node. n = 12/group.

We determined the levels of Th17 and Treg in second lymphoid organs, the spleen and mesenteric lymph node (MLN), by fluorescence-activated cell sorting (FACS). In the spleen, the population of CD4^+^Foxp3^+^ Treg cells slightly decreased and that of CD4^+^IL-17^+^ Th17 cells increased from 8 weeks after surgery. This trend was strongly evident at 12 weeks post-OVX (Figure 2A–2D, Supplementary Figures 3, 4). The Th17-biased development was distinctly detected in the MLN. Moreover, the increase in the population of Th17 cells was consistent with the elevated levels of IL-17 in the circulation (Figure 2E). IL-10 level was reduced from 8 weeks post OVX (Figure 2F).

Taken together, OVX causes bone loss, accompanied with a Th17-enriched and Treg-deficient environment in lymphoid organs. This phenomenon was likely to be initiated at 8 weeks post-surgery.

### OVX-induced vascular impairment precedes bone loss

Osteoporosis is closely related to the impairment of vasculature [30]. A recent study suggested that the dysfunction of the vasculature proceeds via bone loss [30–33], and the type H vessel in the BM supports osteogenesis by protecting osteoprogenitors [30]. Xie et al. found that angiogenic factors such as PDGF-BB determine the temporal-spatial vessel formation for new bone development [32]. That is, the intact vasculature and its paracrine potential heavily influence the maintenance of the bone tissue.

**Figure 3:**
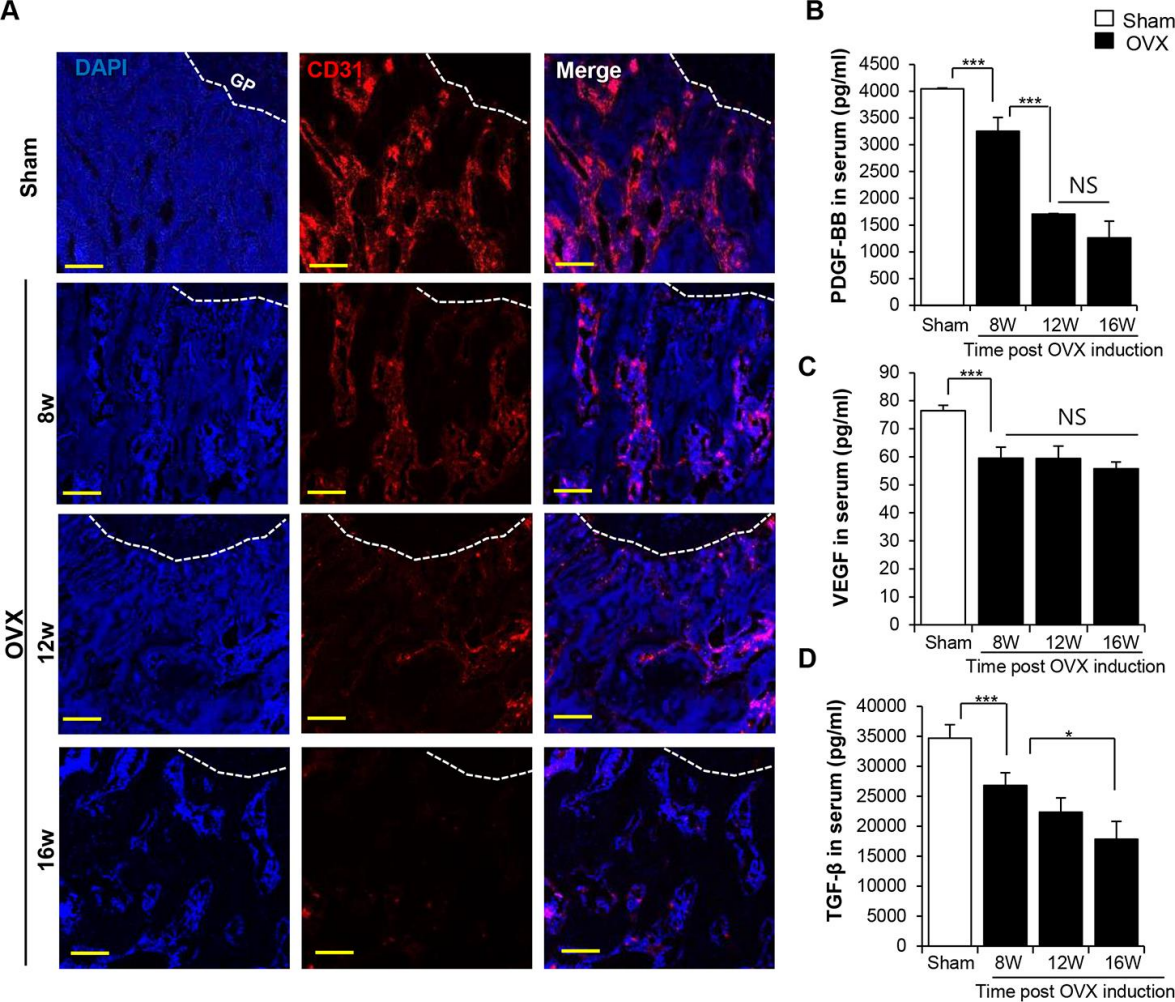
**OVX impairs the vasculature in trabecular bone.** (**A**) Images of immunofluorescence staining for CD31 (red) in distal femoral metaphysis from sham-operation and OVX rat. GP: growth plate. Scale bar: 200 μm. (**B**–**D**) The concentrations of PDGF-BB VEGF and TGF-β in serum were determined by ELISA. p values of less than 0.05 were considered statistically significant (* p <0.05, ** p <0.01, *** p <0.001). n = 12/group.

In the present study, we found that the decrease in the bone density was associated with the decrease in the density of the CD31^+^ type H vessel in the trabecular bone (Figure 3A). Analysis of angiogenic factors in the serum revealed a sharp decline in PDGF-BB, VEGF, and TGF-β levels after OVX in a time-dependent manner (Figure 3B–3D). This observation was consistent with that previously report to prove the relationship between osteoporosis and deficiency of vessels [31].

To analyze the progression of vascular damage, we examined density of the CD31^+^ type H vasculature at 4 weeks post-OVX and found that it was seldom observed in the trabecular bone even at 4 weeks post-OVX, the time corresponding to that without bone loss and systemic inflammation (Supplementary Figures 1 and 2). Moreover, serum VEGF and PDGF-BB levels already dropped at 4 weeks in OVX rats as compared with those observed in control rats (Supplementary Figure 5).

Together these observations corroborate that the deficiency of estrogen after OVX deteriorates the vasculature and its paracrine potential, consequently leading to the loss of bone density.

### SP prevents bone loss by regulating angiogenic and inflammatory responses

**Figure 4:**
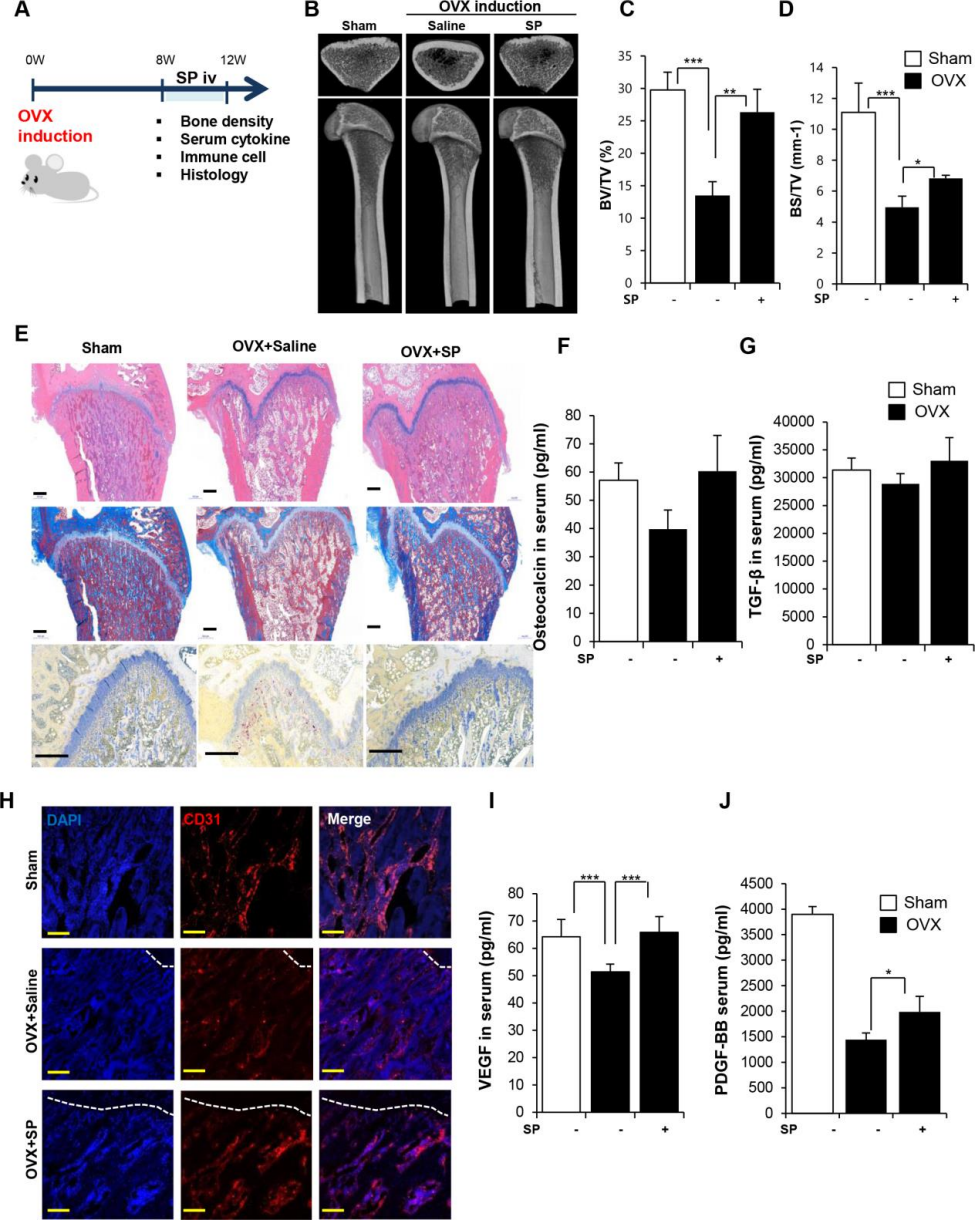
**SP ameliorates bone loss, preserving vasculature and angiogenic factors.** (**A**) Experimental schedule for OVX and SP treatment. (**B**) Representative femoral μCT images. (**C**, **D**) Quantitative analyses of the trabecular bone fraction of femora from rats. (**E**) H&E, Masson’s Trichrome and TRAP staining of distal femoral metaphysis regions. Scale bar: 500 μm. (**F**, **G**) The level of osteocalcin and TGF-β in the blood was elucidated by ELISA. (**H**) Images of immunofluorescence staining for CD31 (red) in distal femoral metaphysis from rat after sham-operation, OVX and OVX with SP treatment. GP: growth plate. Scale bar: 200 μm. (**I**, **J**) The concentration of VEGF and PDGF-BB in the blood was analyzed by ELISA. BV: trabecular bone volume. TV: tissue volume. BS: bone surface. p values of less than 0.05 were considered statistically significant (* p <0.05, ** p <0.01, *** p <0.001. n = 12/group

We have attempted to explore the therapeutic effect of SP on osteoporosis and identified the starting point of bone loss in OVX rats. We administered rats with SP at 8 weeks after surgery, which is responsible for the impairment of both the bone density and vasculature. The efficacy of SP was analyzed at 12 weeks post-surgery (Figure 4A).

Cyclic injection of SP prevented the reduction in bone density, as observed by micro-CT (Figure 4B–4D). Histological analysis revealed the ability of SP injection to decrease the accumulation of the fat tissue and reduce the population of TRAP^+^ activated osteoclasts in the femur (Figure 4E). Serum OCN and TGF-β levels reduced after OVX but these levels recovered after SP treatment (Figure 4F and 4G). SP effect was abrogated by NK-1R antagonist (Supplementary Figure 6).

The vasculature was found to be extremely impaired at 8 weeks post-OVX (Figure 4H), and SP injection could repair the vessel density in the BM to some extent. Serum VEGF and PDGF-BB levels reliably increased after SP treatment as compared with those observed after saline treatment (Figure 4I and 4J).

**Figure 5:**
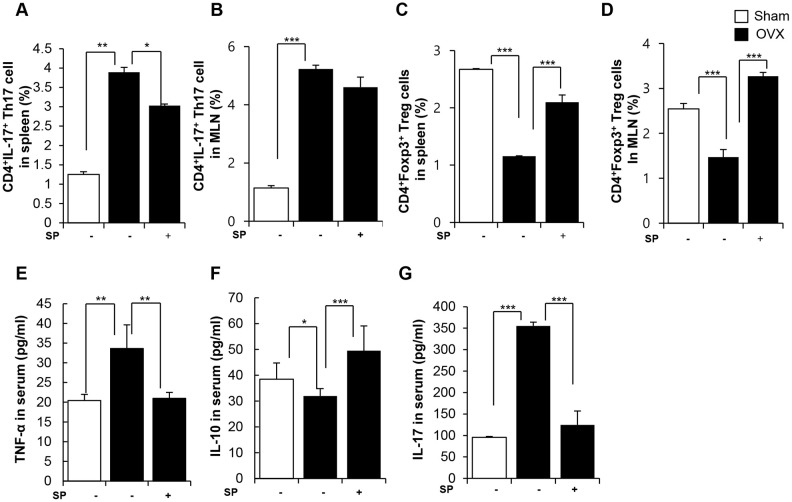
**SP facilitates the development of Treg under osteoporosis condition.** The ratios of CD4^+^IL17^+^ cells in splenocytes (**A**) and in MLN cells (**B**) were analyzed by FACS analysis. The ratios of CD4^+^Foxp3^+^ cells in splenocytes (**C**) and in MLN cells (**D**) were quantified by FACS analysis. (**E**–**G**) The concentrations of TNF-α, IL-10 and IL-17 in serum were analyzed by ELISA. p values of less than 0.05 were considered statistically significant (* p <0.05, ** p <0.01, *** p <0.001). MLN: mesenteric lymph node. n = 12/group.

SP treatment obviously decreased the pool of Th17 cells and enriched Treg cells in both the spleen and MLNs at 12 weeks post-surgery (Figure 5A–5D). While OVX increased TNF-α/IL-17 levels and reduced IL-10 level in the serum, SP treatment reversed the OVX-induced inflammation by decreasing TNF-α/IL-17 and elevating IL-10 levels (Figure 5E–5G).

Thus, OVX develops a pro-inflammatory environment by modulating the profile of immune cells, accompanied by vascular damage. This condition eventually provokes the activation of osteoclasts and loss of bone. However, SP injection could promote Treg generation, rather than Th17, and reduce vascular damage under OVX-induced pathological conditions. These functions of SP may alleviate the reduction in bond density to maintain the intactness of the bone tissue.

### SP is indirectly involved in the development of Treg cells

SP treatment prevented loss of bone density, reduced inflammation, and exhibited high angiogenic potential in vivo. SP was previously reported to induce immune suppression by controlling the profile of immune cells [35–37, 43].

**Figure 6:**
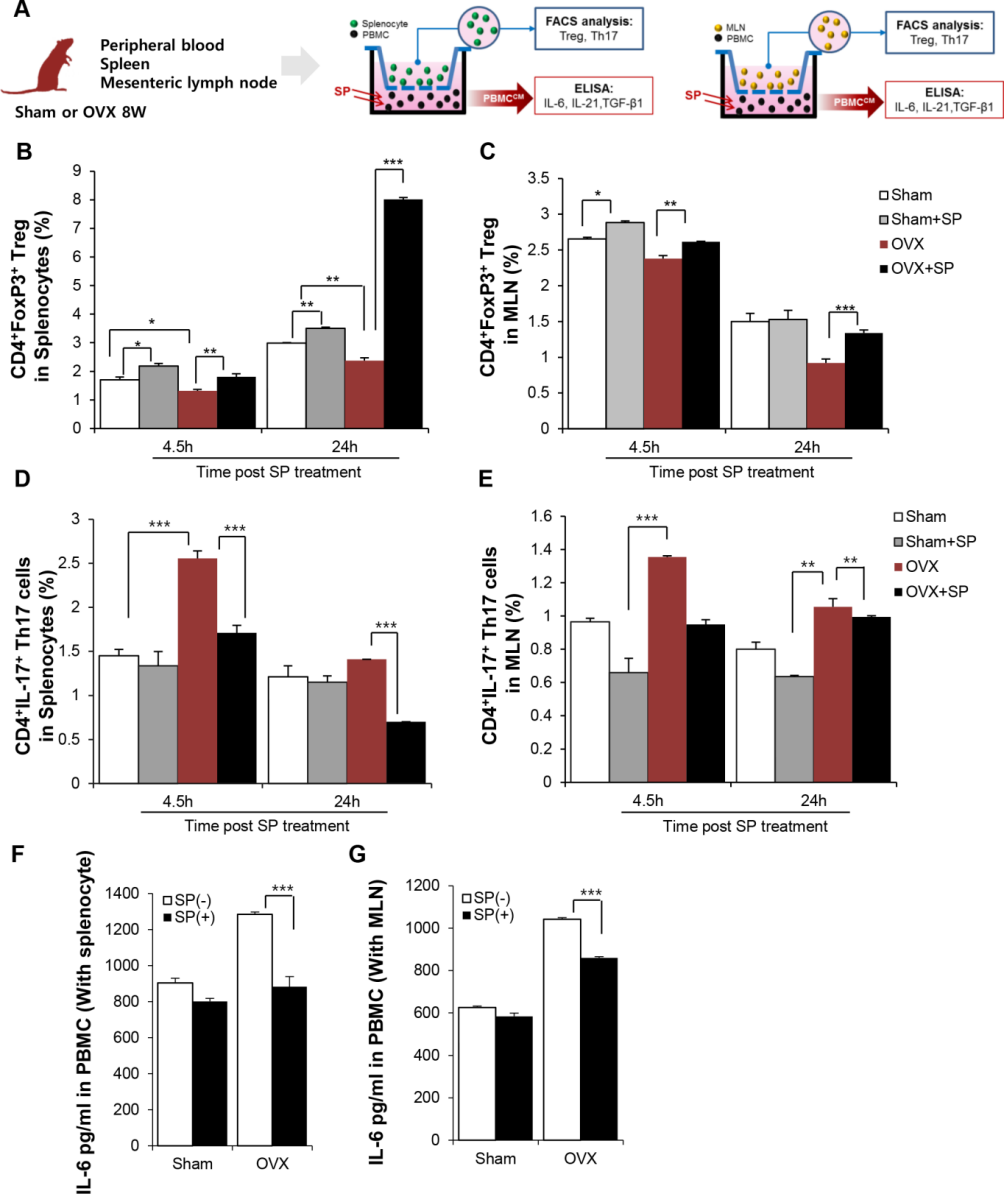
**SP can enrich Treg pool in lymphoid organ by suppressing IL-6 secretion from PBMC.** (**A**) Experimental scheme for coculture of PBMC and splenocyte/MLN. SP was added to PBMC (lower part) and 4.5/24h later, spleen/MLN (Upper) was examined by FACS. The portions of CD4^+^Foxp3^+^ cells in splenocytes (**B**) and in MLN (**C**) were analyzed by FACS post SP treatment PBMC. The ratio of CD4^+^IL17^+^ cells (**D**) in splenocytes and (**E**) in MLN cells was analyzed by FACS. (**F**–**G**) The level of IL-6 in PBMC-conditioned medium with SP was analyzed by ELISA. p values of less than 0.05 were considered statistically significant (* p <0.05, ** p <0.01, *** p <0.001). The data are expressed as the mean ± standard deviation (SD) of three independent experiments. MLN: mesenteric lymph node. n = 4/ experimental setting

To clarify the effect of SP on Treg development under osteoporosis, in vitro co-cultures of peripheral blood mononuclear cells (PBMCs) and spleen and MLN cells from sham or OVX rats were performed (Figure 6A). To mimic the in vivo systemic SP treatment, PBMCs were treated with SP and provide indirect effects of SP on the spleen or MLN cells through blood.

After 4.5 and 24 h, the ratio of Treg and Th17 cells in the spleen/MLN cells was analyzed by FACS. Osteoporosis condition maintained lower number of CD4^+^Foxp^+^ Treg but higher proportion of CD4^+^IL-17^+^ Th17 cells as compared to sham treatment (Figure 6B–6E). However, the level of Treg greatly increased and Th17 pool decreased after SP treatment; this effect was maintained for 24 h. Thus, SP-stimulated PBMCs may be involved in Treg development in lymphoid organs, thereby producing an immunosuppressive environment in osteoporosis (Figure 6B and 6C).

Differentiation of naïve T cells to Tregs or Th17 occurs in an exclusive way. Naïve T cells can differentiate into Tregs or Th17 cells, and the factors that drive differentiation of native T cells into Th17 cells, including IL-6, TGF-β, and IL-21, are well known. The analysis of the conditioned medium of PBMCs showed that the PBMCs from OVX rats had higher levels of IL-6, IL-21, and TGF-β than those from sham rats, indicative of a favorable environment for the generation of Th17. However, SP treatment decreased IL-6 levels (Figure 6F and 6G) and rarely affected TGF-β and IL-21 levels (Supplementary Figure 7).

Thus, this data implies that SP could facilitate the differentiation of naïve T cells into Tregs, but not Th17 cells, by decreasing IL-6 levels in the circulation under osteoporotic stress.

### SP can restore the osteoporosis-mediated impaired activity of stem cells

**Figure 7:**
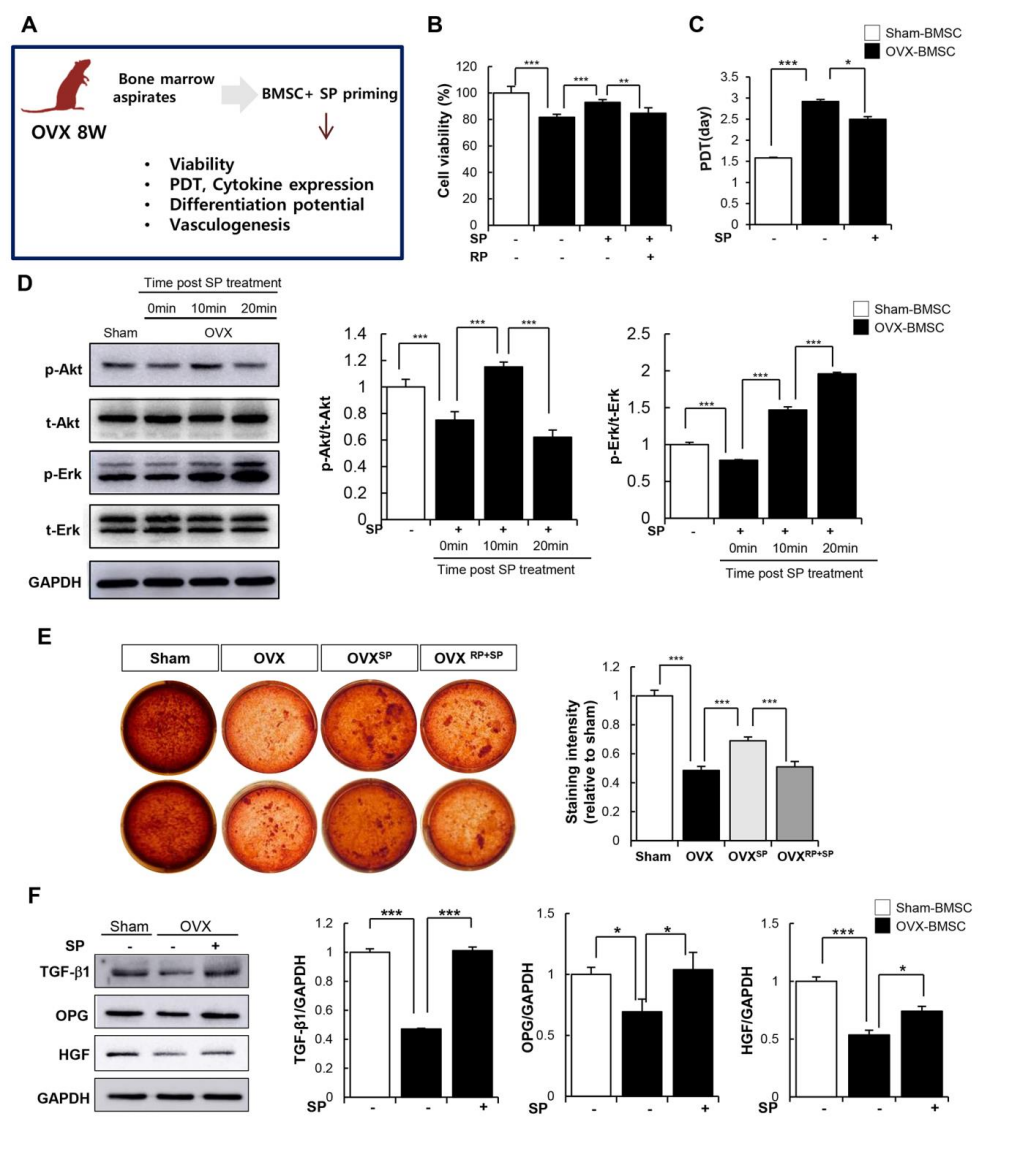
**SP can restore impaired function of BMSC due to osteoporosis.** (**A**) Experimental scheme for direct actions of SP on BMSC in vitro. (**B**) Cell viability was measured by WST-1 assay. (**C**) Population doubling time (PDT) of BMSC was calculated. (**D**) p-Akt, t-Akt, p-Erk and t-Erk protein levels were determined by western blot and their expression levels were quantified using the Image J program, relative to the GAPDH. (**E**) Osteogenic differentiation was exerted by BMSCs and determined by alizarin red staining. (**F**) TGF-β1, OPG and HGF protein expressions in BMSC were elucidated by western blots and quantified relatively. p values of less than 0.05 were considered statistically significant (* p <0.05, ** p <0.01, *** p <0.001). The data are expressed as the mean ± standard deviation (SD) of three independent experiments. OPG: osteoprotegerin, HGF: hepatocyte growth factor. n = 5/ experimental setting.

In addition to its anti-inflammatory function, SP is capable of restoring the impaired activity of BMSCs mediated by inflammation, oxidative stress, and senescence [40, 41]. We investigated whether SP can recover the cellular activity of BMSCs from OVX. BMSCs from OVX 8W were cultured and primed with SP once a day for 2 days in vitro. The effects of SP on the viability, paracrine function, and differentiation potential of BMSCs were evaluated (Figure 7A). Cell viability and repopulation rate were clearly impaired after osteoporosis but could be restored by SP treatment (Figure 7A, 7B). The protein kinase B (AkT) and extracellular signal-regulated kinase (Erk) signaling pathway is highly associated with cell proliferation and survival. As predicted, active Akt and Erk levels reduced after osteoporosis, but this signaling pathway was activated by SP within 10 min (Figure 7D–7F), which probably contributes the improvement in the viability and repopulation of BMSCs.

OVX conditions impair the differentiation ability of BMSCs into osteoblasts. However, SP-primed MSCs showed better osteogenic differentiation and calcium deposition (Figure 7E). TGF-β, OPG, and hepatocyte growth factor (HGF) required for bone formation were elevated after SP treatment (Figure 7F).

**Figure 8:**
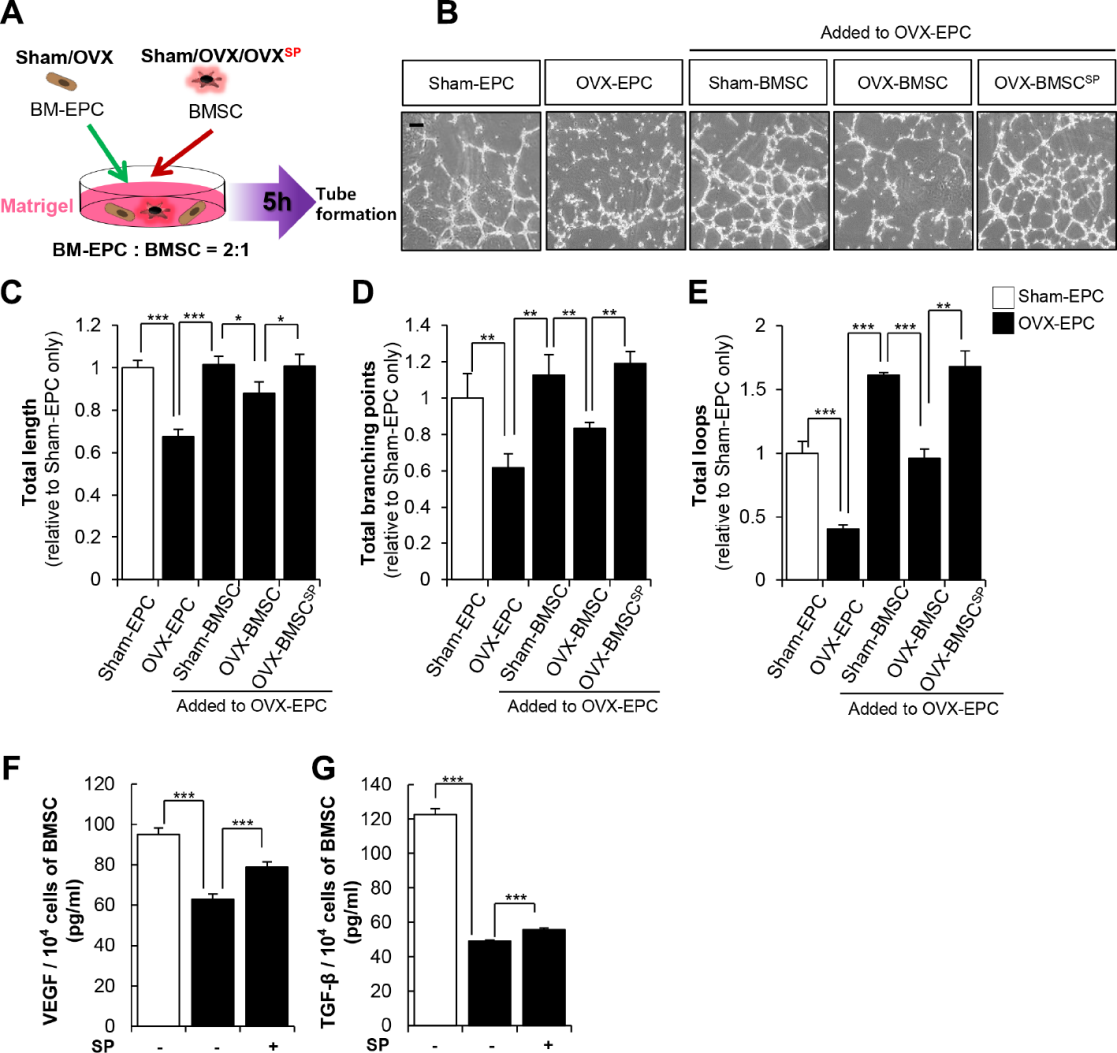
**SP enhances the vascular potential of BMSC from OVX.** (**A**) BMSC and EPC from Sham/OVX rat was primed with SP and then, subjected to vascular tube formation assay. EPC was seeded with or without BMSC on the Matrigel and tube formation ability was observed after 5 hours (**B**) Representative images of tube formation assay on Matrigel in EPC co-cultured with or without BMSC. Scale bar: 200 μm. (**C**–**E**) The total tube length, total branching points and total loops were quantified relatively. (**F**–**G**) The concentrations of VEGF and TGF-β in conditioned medium of BMSCs were measured by ELISA and adjusted to per 1 ^×^ 10^4^ cells. p values of less than 0.05 were considered statistically significant (* p <0.05, ** p <0.01, *** p <0.001). The data are expressed as the mean ± standard deviation (SD) of three independent experiments. n = 5/ experimental setting.

SP treatment restored the BM vasculature in OVX rats. BMSCs constitutively secrete angiogenic factors such as VEGF and TGF-β, contributing to the maintenance and homeostasis of the vasculature in the BM microenvironment. To determine the supportive role of BMSCs in vessel formation, endothelial progenitor cells (EPCs) from OVX rats were co-cultured with BMSCs in Matrigel in vitro (Figure 8A).

As predicted, the EPCs from sham (sham EPC) showed a tubular structure, whereas those from OVX (OVX EPC) failed to develop any vascular structure. However, the coexistence of sham BMSCs enabled the formation of a continuous vasculature in OVX EPCs. To mimic in vivo conditions, OVX BMSCs were primed with SP (OVX-BMSC^SP^) and then incubated with OVX EPCs in Matrigel. OVX BMSCs rarely provided a supportive role in OVX EPC–induced vessel formation. In contrast, OVX-BMSC^SP^ clearly facilitated OVX EPC–induced vessel formation (Figure 8B–8E).

The formation of vasculature is governed by the profile of paracrine factors; thus, SP was thought to promote the production of angiogenic factors in OVX BMSCs. The effect of SP on the secretion of cytokines by OVX BMSCs was assessed through enzyme-linked immunosorbent assay (ELISA). SP restored the levels of VEGF and TGF-β, which were reduced by OVX (Figure 8F and 8G). Thus, SP treatment may enhance the vascular activity of BMSCs possibly by modulating their paracrine action.

Collectively, SP could restore the cellular functions of BMSCs injured by osteoporosis and thus, maintain the pool of BMSCs and osteogenic progenitor cells with vascular potential. These effects may negatively affect the progression of bone loss in vivo.

### Efficacy of SP is superior to that of parathyroid hormone (PTH) therapy

Conventional therapy for osteoporosis includes the administration of calcium, bisphosphonate, or PTH. Of these, PTH is used as a bone-forming agent that necessitates repeated injections once a day for several months. However, PTH exerts opposite effects depending on dosage and is ineffective on the old population including daily alcohol consumption or bisphosphonate taker; thus, it is important to control PTH dosing by limiting injection period or determining the patient population. Despite the associated risks, PTH is the most widely used drug for osteoporosis treatment.

We compared the effect of SP and PTH therapy in OVX rats. According to the application route in the clinic, PTH was subcutaneously injected once a day for 5 days and SP was intravenously injected twice a week from 8 weeks post-OVX.

**Figure 9:**
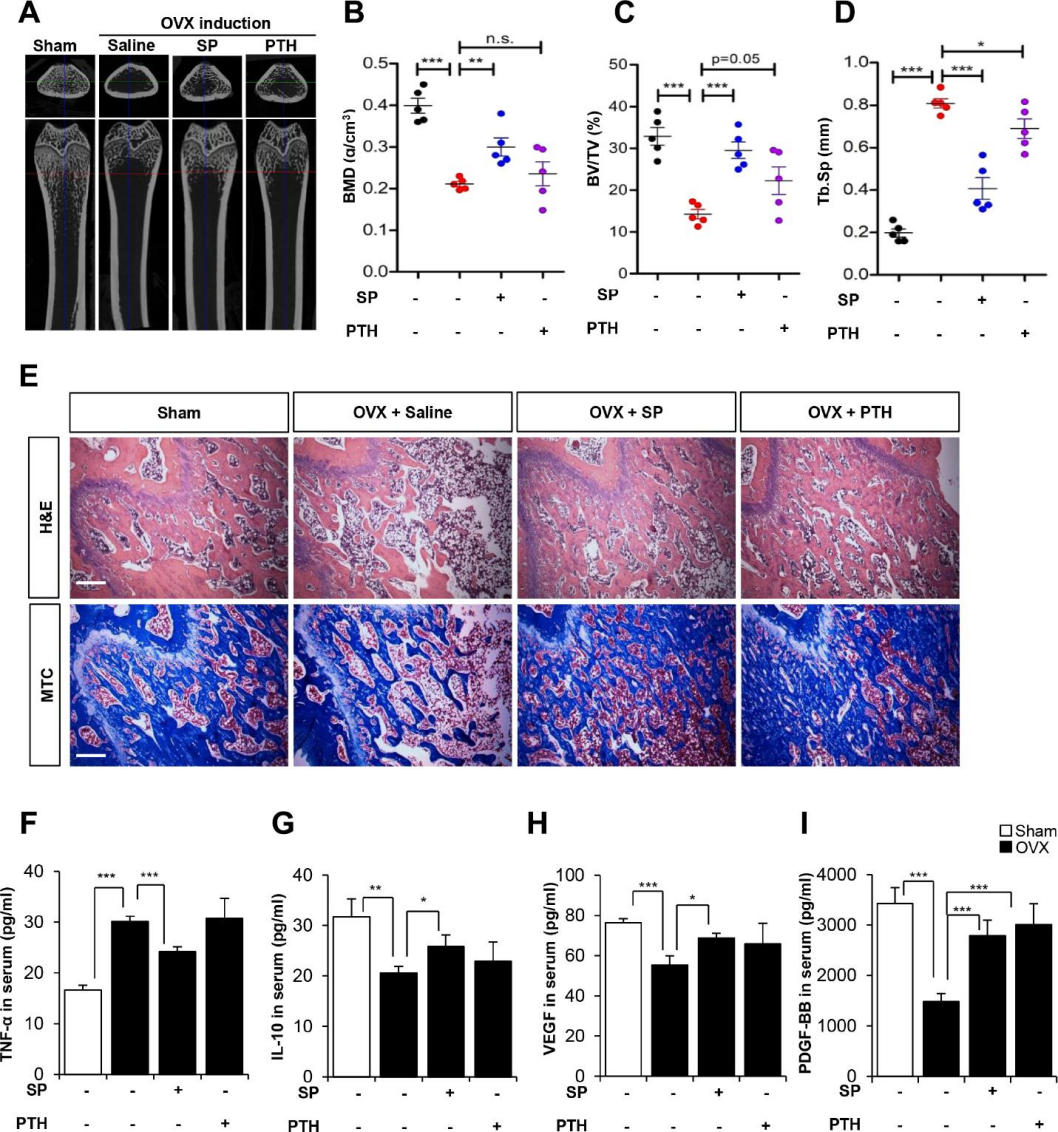
**The efficacy of SP is superior to PTH therapy.** (**A**) Representative femoral μCT images from each group. (**B**–**D**) Quantitative analyses of the trabecular bone fraction of femur from rats. (**E**) H&E and Masson’s Trichrome staining of distal femoral metaphysis regions. Scale bar: 100 μm. (**F**–**I**) The concentration of TNF-α, IL-10, VEGF and PDGF-BB in serum were analyzed by ELISA. BMD: bone mineral density. BV: trabecular bone volume. TV: tissue volume. Tb.Sp: trabecular bone spacing. p values of less than 0.05 were considered statistically significant (* p <0.05, ** p <0.01, *** p <0.001). n = 6/ group.

The analysis of bone density and histology showed that SP injection inhibited bone loss, consistent with previous data. Although PTH tended to exert inhibitory effects on bone loss, these effects were small and showed variations within experimental animals (Figure 9A–9E).

Serum cytokine analysis revealed that SP could reduce TNF-α and restore IL-10, whereas PTH did not suppress inflammatory factors as compared with saline treatment. Angiogenic factors, VEGF and PDGF-BB were declined by OVX, which were restored by SP or PTH treatment. PTH therapy was involved in angiogenic potential, rather than systemic inflammation.

These observations suggest that PTH treatment could prevent bone loss, but its effects were ambiguous. SP injection could inhibit bone loss and facilitate anti-inflammatory and angiogenic conditions. We injected SP twice a week, while PTH treatment was performed for 5 days a week. Considering the treatment schedule and the reported therapeutic effects, SP is expected to have superior performance to PTH therapy.

## DISCUSSION

The prevalence of osteoporosis continues to escalate with the increase in the aging population. Osteoporosis is a complicated disease characterized with the impairment of immune response, vasculature, and stem cell function. Therefore, targeting a cell or molecule alone may be insufficient for the prevention of osteoporosis—a common shortcoming related to conventional drugs such as PTH or bisphosphonate.

SP is known to suppress highly activated immune response [35–37, 43], protect the vascular endothelium [42], and restore stem cell functions [40, 41] under disease conditions. These functions were proven in different non-clinical disease models. Considering these reported functions of SP, we anticipated its inhibitory effect on osteoporosis in vivo.

Prior to SP treatment, we performed immunological analysis during the progression of osteoporosis and found significant differences in the distribution of vasculature, Th17/Treg cell pool, and serum cytokines after OVX. Deficiency of type H vessels in BM was distinct at early time points after OVX when bone loss was not observed, indicating that vascular impairment occurs before the inception of bone loss. Thus, the evaluation of vascular functions may serve as a criterion to determine the risk of osteoporosis prior to the development of severe bone loss.

SP treatment was initiated at 8 weeks post-OVX in response to a serious situation accompanied with vascular damage, bone loss, and inflammation. Treatment with SP for 4 weeks clearly ameliorated the decrease in bone density and facilitated T cell differentiation into Tregs, consistent with the low level of IL-17 and high level of IL-10. These environments could block the activation of osteoclasts and protect BMSCs and osteoblasts in OVX rats. Along with the recovery in bone density, SP prevented the OVX-mediated loss of type H vasculature in BM; this effect may increase the production of angiogenic factors, including VEGF and PDGF-BB and protect osteoprogenitor cells.

To clarify the action of SP at the cellular level, an ex vivo co-culture system of PBMCs and lymphoid cells from OVX was established and the effect of SP was analyzed. OVX rats showed an increase in the population of Th17 and a decrease in Treg cell pool in lymphoid organs as compared with sham control rats. However, the stimulation with SP via PBMCs reversed the ratio of Th17/Treg, thereby creating an immune-suppressive condition.

In addition to T lymphocytes, the activity of OVX BMSCs was also restored by SP treatment, as evident from the increase in cell repopulation, differentiation potential, and cytokine production in vitro. The recovered function of OVX BMSCs by SP directly mediated osteogenesis, thereby suppressing bone loss. SP treatment also reinforced the supportive role of BMSCs during vessel formation, which may have contributed to the retrieval of CD31^+^ vasculature in the BM in osteoporosis.

A comparative study of SP and PTH revealed the superiority of SP treatment over PTH therapy in terms of bone recovery effect and its consistency. PTH targets the repopulation of osteoblasts, while SP exerts multiple effects to prevent bone loss. Thus, it may be difficult to compare the two drugs. However, we could infer the possibility of using SP as an alternative therapy for PTH.

In summary, our data clearly demonstrate the modulation in the immune response and stem cell activity mediated by SP that influenced the progression of osteoporosis in a positive way. The early protection of vasculature and immune suppression may occur in osteoprogenitors or BMSCs to facilitate their survival or proliferation. This is thought to be the key mechanism to prevent the loss of the bone tissue. We have previously reported the efficacy of SP, but this is the first study to analyze its effects in vivo and in vitro on immune response, vasculature, and stem cells. The dose and injection schedule of SP adopted herein were proved nontoxic, as evident from the safety test of SP in GLP [52, 53]. SP-mediated benefits observed in this study are not limited to skeletal diseases such as osteoporosis. Inflammation and dysfunction of stem cell activity are commonly observed in many critical/lethal diseases. Thus, the application of SP can be extended to mediate therapeutic effects in different diseases.

Osteoporosis is known to decrease the expression of SP and elevate NK-1R levels in the BM environment, but not the blood [45, 46], as confirmed in the present study (Supplementary Figure 8). Thus, we suggest relevance of SP injection to osteoporotic stress and anticipate that SP administration was likely to compensate for the deficiency of SP in OVX rats. However, the specific regulation of endogenous SP during bone loss should be explored in future studies.

## MATERIALS AND METHODS

### Induction of osteoporosis animal model

Seven-week-old Sprague Dawley (SD) rats (160-170 g, female) were purchased from DBL (Daehan Bio Link, Seoul, Korea). All animals were maintained under a regular light/dark illumination cycle in an animal holding room and permitted to adapt new environment for 7 days before the experiments. All animals received standard chow diet, and this study was approved by the Ethical Committees for Experimental Animals of Kyung Hee University Hospital with the approval number of KHMC-IACUC 2018-40.

To induce an osteoporosis disease model, female SD rats were anesthetized during the operation by using intraperitoneal injections of Ketamine (100 mg/kg, Yuhan, Seoul, Korea) and Rompun (1.2 mg/kg, Bayer Healthcare, Kyunggi-do, Korea). About 2 cm-long skin incisions was induced both sides of abdomen to expose the dorsolateral abdominal muscles and reached to the peritoneal cavity, then the ovaries were found below the kidney. After identifying the ovary, both the terminologia anatomica and ovarian artery were carefully uncovered and ligated with 5-0 silk and subsequently the ovaries were removed. After hemostasis process, both two sides of abdominal incisions were sutured with 3-0 silk. All of the surgical procedures were performed under sterilized condition. All rats were randomly divided into five groups: (1) Sham, (2) OVX + saline, (3) OVX + SP, (4) OVX + RP67580 (NK-1R antagonist) + SP and (5) OVX + PTH.

### Administration of SP, RP67580 and hPTH (1-34)

SP (Sigma-Aldrich, St. Louis, MO, USA) was diluted in saline (JW Pharmaceuticals, Seoul, Korea) just before use and administrated intravenously twice a week at a dose of 5 nmol/kg (n = 12/group). Saline was used for the vehicle treatment (n = 12/group). RP67580 (TOCRIS, Bristol, UK, NK-1R antagonist) was diluted in saline and administrated intravenously just 5 minutes before SP injection at a dose of 10 nmol/kg (n = 6). Human parathyroid hormone-(1-34) (h-PTH (1-34) (Mimotopes, Melbourne, Australia), was also diluted in saline and administrated subcutaneously five times a week at a dose of 80 μg/kg

### Ex-vivo micro-computed tomography (μ-CT) imaging

Femur was scanned on a μ-CT scanner (SkyScan 1173 X-ray microtomography; Bruker) at a resolution of 13.85 μm to evaluate the degree of osteoporosis. After three-dimensional reconstruction, the bone mineral density (BMD), the bone volume fraction (BV/TV) and the trabecular bone space (Tb.sp) in the metaphysis area were used to calculate using CT analysis software. (n = 12/group).

### Enzyme-linked immunosorbent assay (ELISA)

Cytokine profiles in serum, bone marrow aspirates and conditioned medium were analyzed by ELISA. The concentrations of TNF-α (Biolegend, San Diego, CA, USA), IL-6, IL-10 (R&D system), IL-21 (LSBio, Seattle, WA, USA), PDGF-BB, VEGF, TGF-β (R&D system, #MB100B), Glu-osteocalcin (OCN) (Takara, Shiga, Japan), C-telopeptide of type 1 collagen (CTX) and osteoprotegerin (OPG) Cusabio) in serum or conditioned medium samples were measured by ELISA according to the manufacturer’s instructions. In brief, all reagents, standard dilutions, and samples were prepared and added to primary antibody-coated well as directed for 2h at room temperature (RT). Next, the horseradish peroixdase-conjuated secondary antibody solution was treated to each well. 1 or 2 hours later, substrate solution was added. Once the color of the solution changed to blue, the reaction was stopped, and the optical density was measured with the wavelength correction set to 450 nm using an EMax Endpoint ELISA Microplate Reader (Molecular Devices, Sunnyvale, CA).

### Histological analysis

Femur was isolated and fixed in 3.7 % paraformaldehyde (Sigma-Aldrich) for 1 day. Samples were decalcified in decalcifying solution (Sigma-Aldrich) and processed with a TP1020 tissue processor (Leica Biosystems, Wetzlar, Germany) to make paraffin blocks, and 5.0-μm-thick sections were prepared.

For hematoxylin and eosin (H&E) staining, paraffin-sectioned samples were hydrated in alcohol (Merck, Germany). After washing in tap water, nuclei were stained with hematoxylin (Sigma-Aldrich) for 10 minutes and washed in tap water. To stain the cytoplasm, eosin Y (Sigma-Aldrich) was applied for 10 seconds and the samples were washed with tap water and then mounted.

Trichrome staining was performed using the NovaUltraTM Masson Trichrome Stain Kit (IHC World, Woodstock, MD). Staining for tartrate-resistant acid phosphatase (TRAP) was carried out by TRAP staining kit (Sigma Aldrich, Saint Louis, MI, USA).

For immunofluorescence staining, rehydrated samples were boiled with 0.01 M sodium citrate for antigen unmasking and then treated with 0.1 % sodium borohydride (Sigma-Aldrich) to quench activity of endogenous fluorescence and permeabilized with 0.3 % Triton-X 100. Nonspecific binding was blocked by incubating the samples with 2 % normal horse serum for 1 hour at RT. Primary antibodies against CD31 (Abcam) were treated overnight. After washing with phosphate-buffered saline for three times, the samples were incubated with a Cy3-conjugated secondary antibody (Jackson ImmunoResearch Laboratories) for 1 hour at RT. After washing, the samples mounted with VectaMount reagent (Vector Laboratories).

### Isolation of splenocytes and MLN cells

The spleen and MLN of SD rat was aseptically isolated and, trimmed of all connective tissues. Single cells of spleen and MLN were obtained by mechanical force with syringe plunger. The cell suspension was centrifuged at 1500 rpm for 5 min and washed in 1 x PBS (Welgene, Daegu, Korea) containing 1 % P/S (Welgene).

### Fluorescence-activated cell sorting (FACS) analysis

To detect CD4^+^Foxp3^+^ Treg cells in the spleen and MLN, 2 × 10^7^ of splenocytes and MLN cells were pretreated by True-Nuclear Transcription Factor Buffer Set (Biolegend, San Diego, CA, USA) and then incubated with allophycocyanin (APC)-conjugated CD4 antibody (Biolegend) and PE-conjugated anti-Foxp3 antibody (Biolegend).

For CD4^+^IL17^+^ Th17 cells in the spleen and MLN, 2 × 10^7^ of splenocytes and MLN cells were incubated with allophycocyanin (APC)-conjugated CD4 antibody (Biolegend) and phycoerythrin (PE)-conjugated anti-CD17 antibody (eBioscience, San Diego, CA, USA).

The fractions of Treg, and Th17 cells from each tissue were analyzed using a FACSCalibur Flow Cytometer using the CellQuest software (Becton Dickinson, San Jose, CA).

### Co-culture of PBMCs with splenocytes or MLN cells

For indirect co-culture of peripheral blood mononuclear cells (PBMCs) and splenocytes or MLN cells, splenocytes or MLN cells (2 × 10^6^) were seeded on upper transwell insert (0.4 μm pore size; Corning, NY, USA) and PBMCs (2 × 10^6^) were seeded on 6 well plate (Corning). SP was added to PBMCs and coculture was maintained for 24h. After termination of coculture, the ratios of both Treg cells and Th17 cells in splenocytes and MLN cells were analyzed by FACS analysis and IL-6, IL-21 and TGF-β1 expression level of PBMCs in each culture supernatant was measured by ELISA.

### WST-1 assay

10 μl of WST-1 (Roche, Indianapolis, IN, USA), solution was added to each well at 10 % the total volume of the medium, and the plate was incubated for 1 hour at 37 °C in 5 % CO_2_. After incubation, the optical density values for each well were measured at a wavelength of 450 nm using an EMax Endpoint ELISA Microplate Reader.

### Osteogenic differentiation of rat BMSCs

Rat BMSCs were seeded at a density of 5 × 10^3^ cells/cm^2^, cultured overnight in mesenchymal stem cell growth basal medium (MSCGM; Lonza, Basel, Switzerland), and then induced to undergo osteogenesis for up to 15 days in rat osteoblast differentiation medium (Amsbio, Cambridge, MA, USA). Matrix mineralization by BMSC-derived osteoblasts was determined by using Alizarin Red S (Sigma-Aldrich), and staining quantified by measuring the optical densities of extracted stain at 560 nm**.**

### Tube formation assay

Matrigel (BD, San Jose, CA, USA) was coated on μ-Slide Angiogenesis (Ibidi, Munich, Germany) and incubated for 30min at 37 °C in 5 % CO_2_. EPCs were incubated on the Matrigel with or without BMSCs for 5 hours at 37 °C in 5 % CO_2_. The tube structure was observed under phase-contrast microscopy. And the quantification of tube formation was analyzed by Image J.

### Preparation of protein extracts and western blot analysis

Protein was extracted by cell lysis buffer (20 mM Tris-HCl (pH=7.5), 150 mM NaCl, 1 mM Na_2_EDTA, 1 mM EGTA, 1 % Triton, 2.5 mM sodium pyrophosphate, 1 mM β-glycerophosphate, 1 mM Na_3_VO_4_ (Cell Signaling Technology, Danvers, MA, USA) and 1 μg/ml leupeptin (Cell Signaling Technology) containing phenylmethylsulfonyl fluoride (Sigma-Aldrich). The supernatants were collected by centrifugation at 12000 rpm for 10 min. The protein concentration was determined using a BCA Protein Assay Kit (Thermo Fisher Scientific, Waltham, MA, USA).

The proteins were denatured and electrophoresed using sodium dodecyl sulfate (SDS)-polyacrylamide gel electrophoresis and transferred to a nitrocellulose membrane. Blocking of the membrane was performed with 5 % skim milk (Bio-Rad) or bovine serum albumin (Sigma-Aldrich) for 1 h. After blocking, the membranes were incubated with the primary antibodies for p-Akt (Cell Signaling Technology), t-Akt (Cell Signaling Technology), p-Erk (Cell Signaling Technology), t-Erk (Cell Signaling Technology), TGF-β1 (Abcam, Cambridge, MA, USA), OPG (Abcam), HGF (Abcam), NK-1R (Novus Biologicals, Littleton, CO, USA) and glyceraldehyde 3-phosphate dehydrogenase (GAPDH) (Abcam), followed by anti-immunoglobulin G horseradish peroxidase-conjugated secondary antibody and visualized with a chemiluminescence substrate (Dogen). Expression level was quantified using the Image J program.

### Statistics

All Data are presented as the mean standard deviation (SD) of three independent experiments. P values of less than 0.05 were considered statistically significant. Statistical analysis of all data was carried out by an unpaired, two-tailed Student t-test.

## Supplementary Material

Supplementary Figures

## References

[1] Sözen T, Özışık L, Başaran NÇ. An overview and management of osteoporosis. Eur J Rheumatol. 2017; 4:46–56. 10.5152/eurjrheum.2016.048 28293453 PMC5335887

[2] Guido G, Scaglione M, Fabbri L. Fragility fractures’ osteosynthesis survey. Aging Clin Exp Res. 2011 (2 Suppl); 23:57–59. 21970925

[3] Tomkinson A, Reeve J, Shaw RW, Noble BS. The death of osteocytes via apoptosis accompanies estrogen withdrawal in human bone. J Clin Endocrinol Metab. 1997; 82:3128–35. 10.1210/jcem.82.9.4200 9284757

[4] Fliefel R, Tröltzsch M, Kühnisch J, Ehrenfeld M, Otto S. Treatment strategies and outcomes of bisphosphonate-related osteonecrosis of the jaw (BRONJ) with characterization of patients: a systematic review. Int J Oral Maxillofac Surg. 2015; 44:568–85. 10.1016/j.ijom.2015.01.026 25726090

[5] Whyte MP, Wenkert D, Clements KL, McAlister WH, Mumm S. Bisphosphonate-induced osteopetrosis. N Engl J Med. 2003; 349:457–63. 10.1056/NEJMoa023110 12890844

[6] Tella SH, Gallagher JC. Prevention and treatment of postmenopausal osteoporosis. J Steroid Biochem Mol Biol. 2014; 142:155–70. 10.1016/j.jsbmb.2013.09.008 24176761 PMC4187361

[7] Fierro FA, Nolta JA, Adamopoulos IE. Concise review: stem cells in osteoimmunology. Stem Cells. 2017; 35:1461–67. 10.1002/stem.2625 28390147 PMC6047890

[8] Brylka LJ, Schinke T. Chemokines in physiological and pathological bone remodeling. Front Immunol. 2019; 10:2182. 10.3389/fimmu.2019.02182 31572390 PMC6753917

[9] Cenci S, Weitzmann MN, Roggia C, Namba N, Novack D, Woodring J, Pacifici R. Estrogen deficiency induces bone loss by enhancing t-cell production of TNF-alpha. J Clin Invest. 2000; 106:1229–37. 10.1172/JCI11066 11086024 PMC381439

[10] Weitzmann MN, Roggia C, Toraldo G, Weitzmann L, Pacifici R. Increased production of IL-7 uncouples bone formation from bone resorption during estrogen deficiency. J Clin Invest. 2002; 110:1643–50. 10.1172/JCI15687 12464669 PMC151629

[11] Wei S, Kitaura H, Zhou P, Ross FP, Teitelbaum SL. IL-1 mediates TNF-induced osteoclastogenesis. J Clin Invest. 2005; 115:282–90. 10.1172/JCI23394 15668736 PMC544608

[12] Zhang Y, Khan D, Delling J, Tobiasch E. Mechanisms underlying the osteo- and adipo-differentiation of human mesenchymal stem cells. ScientificWorldJournal. 2012; 2012:793823. 10.1100/2012/793823 22500143 PMC3317548

[13] Fitzsimmons RE, Mazurek MS, Soos A, Simmons CA. Mesenchymal stromal/stem cells in regenerative medicine and tissue engineering. Stem Cells Int. 2018; 2018:8031718. 10.1155/2018/8031718 30210552 PMC6120267

[14] Chen E, Liu G, Zhou X, Zhang W, Wang C, Hu D, Xue D, Pan Z. Concentration-dependent, dual roles of IL-10 in the osteogenesis of human BMSCs via P38/MAPK and NF-κB signaling pathways. FASEB J. 2018; 32:4917–29. 10.1096/fj.201701256RRR 29630408

[15] Ferrucci L, Fabbri E. Inflammageing: chronic inflammation in ageing, cardiovascular disease, and frailty. Nat Rev Cardiol. 2018; 15:505–22. 10.1038/s41569-018-0064-2 30065258 PMC6146930

[16] Ginaldi L, Di Benedetto MC, De Martinis M. Osteoporosis, inflammation and ageing. Immun Ageing. 2005; 2:14. 10.1186/1742-4933-2-14 16271143 PMC1308846

[17] Li J, Liu X, Zuo B, Zhang L. The role of bone marrow microenvironment in governing the balance between osteoblastogenesis and adipogenesis. Aging Dis. 2015; 7:514–25. 10.14336/AD.2015.1206 27493836 PMC4963194

[18] Manolagas SC. From estrogen-centric to aging and oxidative stress: a revised perspective of the pathogenesis of osteoporosis. Endocr Rev. 2010; 31:266–300. 10.1210/er.2009-0024 20051526 PMC3365845

[19] Sato K, Suematsu A, Okamoto K, Yamaguchi A, Morishita Y, Kadono Y, Tanaka S, Kodama T, Akira S, Iwakura Y, Cua DJ, Takayanagi H. Th17 functions as an osteoclastogenic helper T cell subset that links T cell activation and bone destruction. J Exp Med. 2006; 203:2673–82. 10.1084/jem.20061775 17088434 PMC2118166

[20] Zaiss MM, Axmann R, Zwerina J, Polzer K, Gückel E, Skapenko A, Schulze-Koops H, Horwood N, Cope A, Schett G. Treg cells suppress osteoclast formation: a new link between the immune system and bone. Arthritis Rheum. 2007; 56:4104–12. 10.1002/art.23138 18050211

[21] Tesmer LA, Lundy SK, Sarkar S, Fox DA. Th17 cells in human disease. Immunol Rev. 2008; 223:87–113. 10.1111/j.1600-065X.2008.00628.x 18613831 PMC3299089

[22] Xu S, Cao X. Interleukin-17 and its expanding biological functions. Cell Mol Immunol. 2010; 7:164–74. 10.1038/cmi.2010.21 20383173 PMC4002915

[23] Wei L, Laurence A, Elias KM, O’Shea JJ. IL-21 is produced by Th17 cells and drives IL-17 production in a STAT3-dependent manner. J Biol Chem. 2007; 282:34605–10. 10.1074/jbc.M705100200 17884812 PMC2323680

[24] Liu SM, Lee DH, Sullivan JM, Chung D, Jäger A, Shum BO, Sarvetnick NE, Anderson AC, Kuchroo VK. Differential IL-21 signaling in APCs leads to disparate Th17 differentiation in diabetes-susceptible NOD and diabetes-resistant NOD.Idd3 mice. J Clin Invest. 2011; 121:4303–10. 10.1172/JCI46187 22019586 PMC3204832

[25] Sun L, Fu J, Zhou Y. Metabolism controls the balance of Th17/t-regulatory cells. Front Immunol. 2017; 8:1632. 10.3389/fimmu.2017.01632 29230216 PMC5712044

[26] Wang M, Tian T, Yu S, He N, Ma D. Th17 and treg cells in bone related diseases. Clin Dev Immunol. 2013; 2013:203705. 10.1155/2013/203705 24187560 PMC3800633

[27] Dar HY, Singh A, Shukla P, Anupam R, Mondal RK, Mishra PK, Srivastava RK. High dietary salt intake correlates with modulated Th17-treg cell balance resulting in enhanced bone loss and impaired bone-microarchitecture in male mice. Sci Rep. 2018; 8:2503. 10.1038/s41598-018-20896-y 29410520 PMC5802842

[28] Srivastava RK, Dar HY, Mishra PK. Immunoporosis: immunology of osteoporosis-role of T cells. Front Immunol. 2018; 9:657. 10.3389/fimmu.2018.00657 29675022 PMC5895643

[29] Ansari M. Bone tissue regeneration: biology, strategies and interface studies. Prog Biomater. 2019; 8:223–37. 10.1007/s40204-019-00125-z 31768895 PMC6930319

[30] Kusumbe AP, Ramasamy SK, Adams RH. Coupling of angiogenesis and osteogenesis by a specific vessel subtype in bone. Nature. 2014; 507:323–28. 10.1038/nature13145 24646994 PMC4943525

[31] Wang L, Zhou F, Zhang P, Wang H, Qu Z, Jia P, Yao Z, Shen G, Li G, Zhao G, Li J, Mao Y, Xie Z, et al. Human type H vessels are a sensitive biomarker of bone mass. Cell Death Dis. 2017; 8:e2760. 10.1038/cddis.2017.36 28471445 PMC5520742

[32] Xie H, Cui Z, Wang L, Xia Z, Hu Y, Xian L, Li C, Xie L, Crane J, Wan M, Zhen G, Bian Q, Yu B, et al. PDGF-BB secreted by preosteoclasts induces angiogenesis during coupling with osteogenesis. Nat Med. 2014; 20:1270–78. 10.1038/nm.3668 25282358 PMC4224644

[33] Yin H, Huang J, Cao X, Wang ZX, Cao J, Hu Y, Luo J, Tan YJ, Chen TH, Chen CY, Xie H. Inhibition of src homology 2 domain-containing protein tyrosine phosphatase-2 facilitates CD31hiEndomucinhi blood vessel and bone formation in ovariectomized mice. Cell Physiol Biochem. 2018; 50:1068–83. 10.1159/000494531 30355920

[34] Harrison S, Geppetti P. Substance p. Int J Biochem Cell Biol. 2001; 33:555–76. 10.1016/s1357-2725(01)00031-0 11378438

[35] Hong HS, Son Y. Substance P ameliorates collagen II-induced arthritis in mice via suppression of the inflammatory response. Biochem Biophys Res Commun. 2014; 453:179–84. 10.1016/j.bbrc.2014.09.090 25264193

[36] Jiang MH, Chung E, Chi GF, Ahn W, Lim JE, Hong HS, Kim DW, Choi H, Kim J, Son Y. Substance P induces M2-type macrophages after spinal cord injury. Neuroreport. 2012; 23:786–92. 10.1097/WNR.0b013e3283572206 22825006

[37] Hong HS, Hwang DY, Park JH, Kim S, Seo EJ, Son Y. Substance-P alleviates dextran sulfate sodium-induced intestinal damage by suppressing inflammation through enrichment of M2 macrophages and regulatory T cells. Cytokine. 2017; 90:21–30. 10.1016/j.cyto.2016.10.002 27750083

[38] Hong HS, Lee J, Lee E, Kwon YS, Lee E, Ahn W, Jiang MH, Kim JC, Son Y. A new role of substance P as an injury-inducible messenger for mobilization of CD29(+) stromal-like cells. Nat Med. 2009; 15:425–35. 10.1038/nm.1909 19270709

[39] An YS, Lee E, Kang MH, Hong HS, Kim MR, Jang WS, Son Y, Yi JY. Substance P stimulates the recovery of bone marrow after the irradiation. J Cell Physiol. 2011; 226:1204–13. 10.1002/jcp.22447 20945355

[40] Baek SM, Son Y, Hong HS. Substance P blocks the impairment of paracrine potential of MSC due to long term culture. Mol Cell Toxicol. 2018; 14:283–290. 10.1007/s13273-018-0031-3

[41] Jin Y, Hong HS, Son Y. Substance P enhances mesenchymal stem cells-mediated immune modulation. Cytokine. 2015; 71:145–53. 10.1016/j.cyto.2014.10.003 25461392

[42] Piao J, Hong HS, Son Y. Substance P ameliorates tumor necrosis factor-alpha-induced endothelial cell dysfunction by regulating eNOS expression in vitro. Microcirculation. 2018; 25:e12443. 10.1111/micc.12443 29412499

[43] Kim S, Piao J, Hwang DY, Park JS, Son Y, Hong HS. Substance P accelerates wound repair by promoting neovascularization and preventing inflammation in an ischemia mouse model. Life Sci. 2019; 225:98–106. 10.1016/j.lfs.2019.04.015 30959026

[44] Hong HS, Kim S, Lee S, Woo JS, Lee KH, Cheng XW, Son Y, Kim W. substance-P prevents cardiac ischemia-reperfusion injury by modulating stem cell mobilization and causing early suppression of injury-mediated inflammation. Cell Physiol Biochem. 2019; 52:40–56. 10.33594/000000004 30790504

[45] Liu H, Xiong Y, Wang H, Yang L, Wang C, Liu X, Wu Z, Li X, Ou L, Zhang R, Zhu X. Effects of water extract from epimedium on neuropeptide signaling in an ovariectomized osteoporosis rat model. J Ethnopharmacol. 2018; 221:126–36. 10.1016/j.jep.2018.04.035 29705515

[46] Ding WG, Zhang ZM, Zhang YH, Jiang SD, Jiang LS, Dai LY. Changes of substance P during fracture healing in ovariectomized mice. Regul Pept. 2010; 159:28–34. 10.1016/j.regpep.2009.11.004 19903498

[47] Zhao R. Immune regulation of bone loss by Th17 cells in oestrogen-deficient osteoporosis. Eur J Clin Invest. 2013; 43:1195–202. 10.1111/eci.12158 24033116

[48] Pène J, Chevalier S, Preisser L, Vénéreau E, Guilleux MH, Ghannam S, Molès JP, Danger Y, Ravon E, Lesaux S, Yssel H, Gascan H. Chronically inflamed human tissues are infiltrated by highly differentiated Th17 lymphocytes. J Immunol. 2008; 180:7423–30. 10.4049/jimmunol.180.11.7423 18490742

[49] Acosta-Rodriguez EV, Napolitani G, Lanzavecchia A, Sallusto F. Interleukins 1beta and 6 but not transforming growth factor-beta are essential for the differentiation of interleukin 17-producing human T helper cells. Nat Immunol. 2007; 8:942–49. 10.1038/ni1496 17676045

[50] Kotake S, Sato K, Kim KJ, Takahashi N, Udagawa N, Nakamura I, Yamaguchi A, Kishimoto T, Suda T, Kashiwazaki S. Interleukin-6 and soluble interleukin-6 receptors in the synovial fluids from rheumatoid arthritis patients are responsible for osteoclast-like cell formation. J Bone Miner Res. 1996; 11:88–95. 10.1002/jbmr.5650110113 8770701

[51] Romas E, Gillespie MT, Martin TJ. Involvement of receptor activator of NFkappaB ligand and tumor necrosis factor-alpha in bone destruction in rheumatoid arthritis. Bone. 2002; 30:340–46. 10.1016/s8756-3282(01)00682-2 11856640

[52] Hong HS, Yim SV, Son Y. Genotoxicity studies of substance-P by using short-term assay. Mol Cell Toxicol. 2016; 12:447–52. 10.1007/s13273-016-0049-3

[53] Hong HS, Yim SV, Son Y. Evaluation of substance-P toxicity with single dose and repeated dose in rats. Mol Cell Toxicol. 2015; 11:201–11. 10.1007/s13273-015-0019-1

