# Supplementary material for: Substance P blocks ovariectomy-induced bone loss by modulating inflammation and potentiating stem cell function
Source: Aging (Albany NY). 2020 Oct 27;12(20):20753–77. doi: 10.18632/aging.104008 (PMC7655156; doi:10.18632/aging.104008)
Supplement: Supplementary Figures [file aging-12-104008-s001..pdf]

SUPPLEMENTARY FIGURES

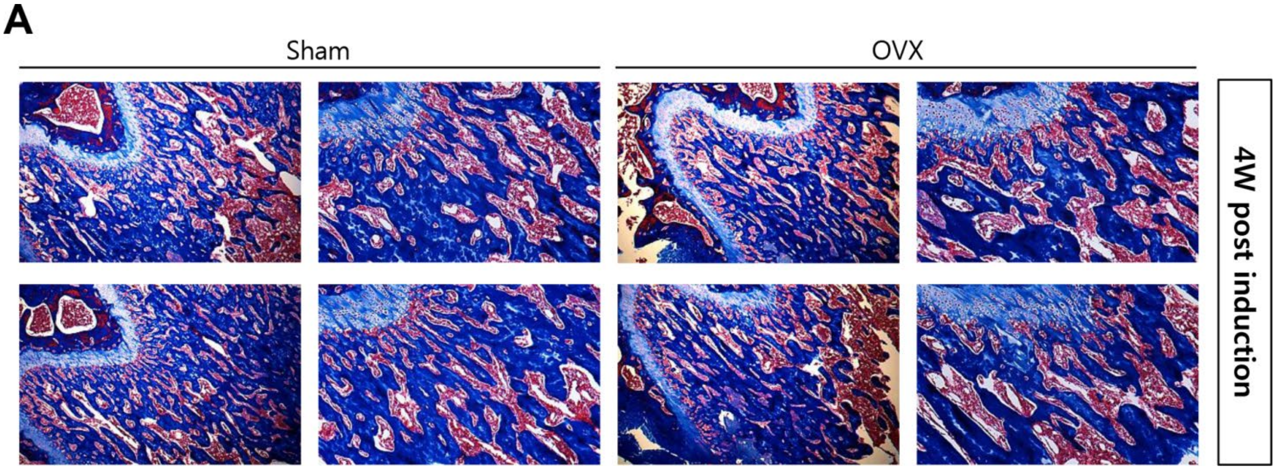

Supplementary Figure 1. OVX does not induce bone loss within 4 weeks. Masson's Trichrome staining of distal femoral metaphysis regions from rats after sham-operation and OVX for 4 weeks. Scale bar: 100 μm.

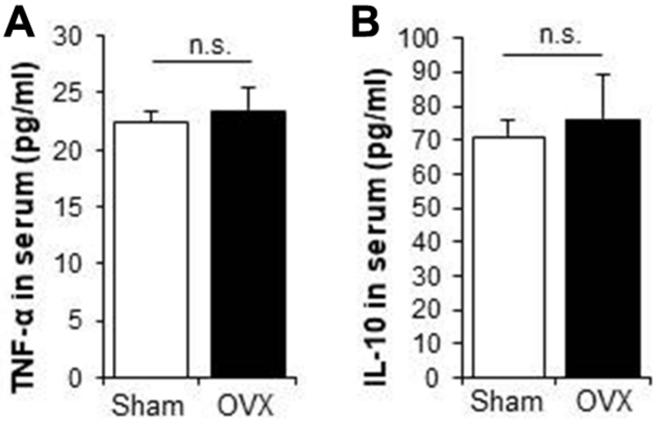

Supplementary Figure 2. OVX can not induce inflammation within 4 weeks. The concentrations of TNF-α (**A**) and IL-10 (**B**) in serum were analyzed by ELISA after 4 weeks of OVX induction and compared with Sham group. *p* values of less than 0.05 were considered statistically significant (* *p* <0.05, ** *p* <0.01, *** *p* <0.001). The data are expressed as the mean ± standard deviation (SD) of three independent experiments.

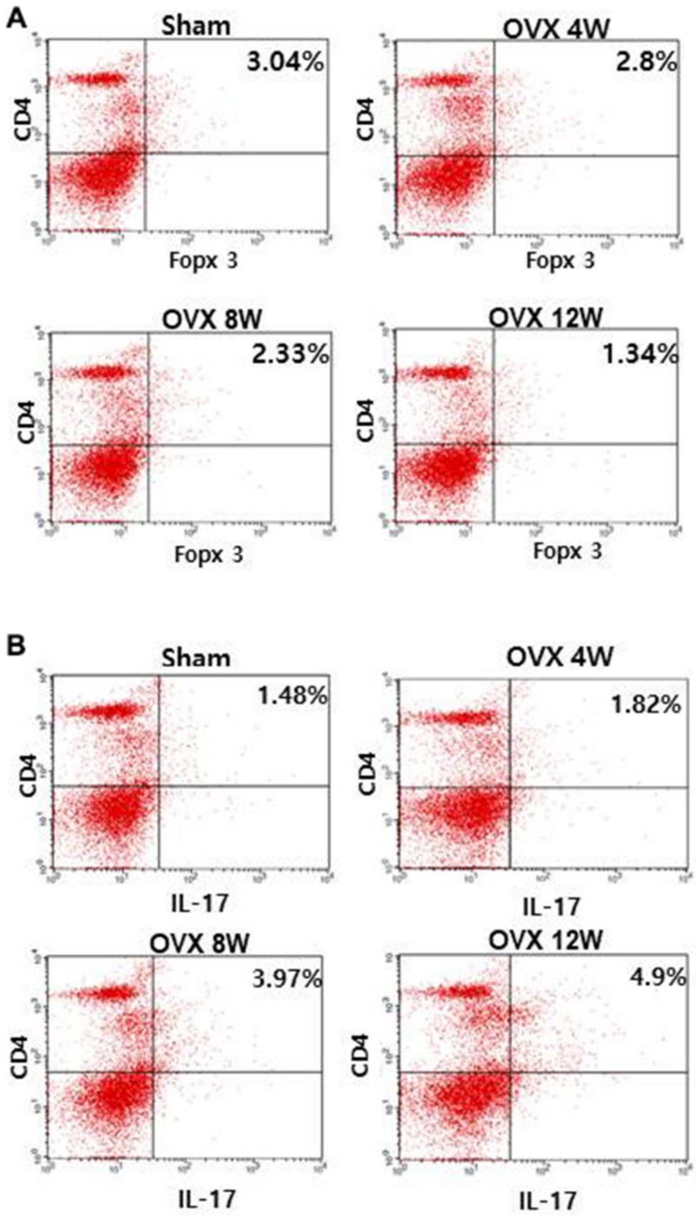

Supplementary Figure 3. OVX reduces Treg cell pools, enriching Th17 cell pools in the spleen. The ratios of (**A**) $CD4^{+}Foxp3^{+}$ cells and (**B**) $CD4^{+}IL17^{+}$ cells in splenocytes were measured by FACS analysis. The data are expressed as the mean ± standard deviation (SD) *p* values of less than 0.05 were considered statistically significant (* p <0.05, ** p <0.01, *** p <0.001). The data are expressed as the mean ± standard deviation (SD).

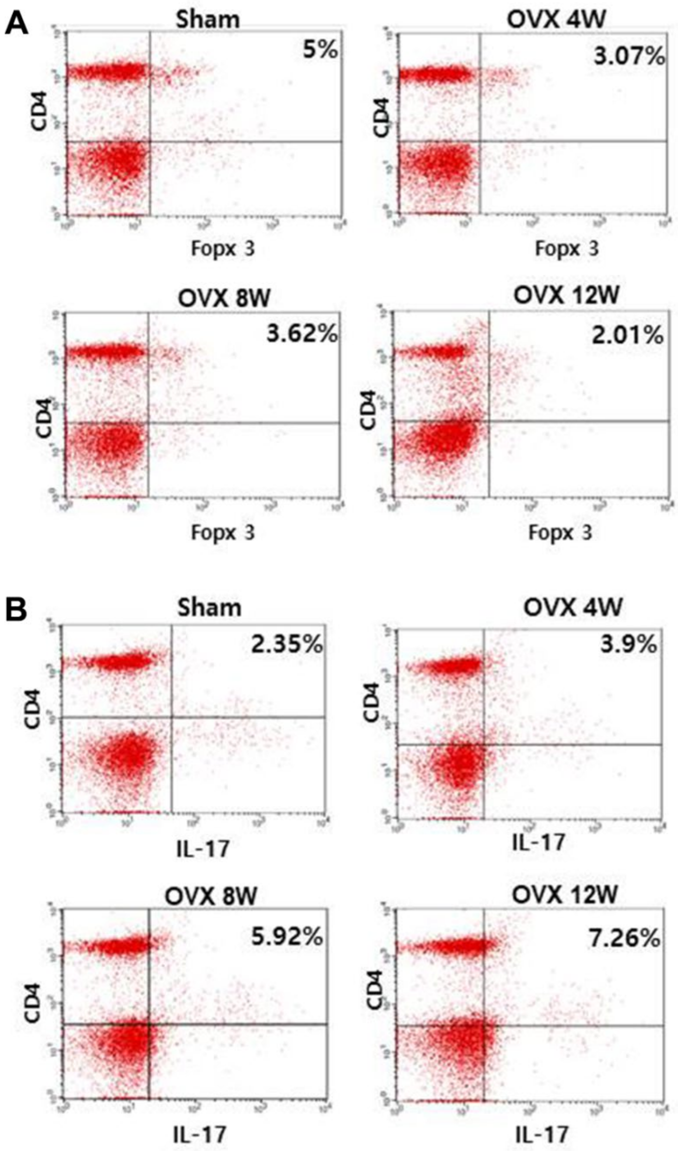

Supplementary Figure 4. OVX declines Treg cell pools but elevates Th17 cell pools in the mesenteric lymph node. The ratios of (**A**) $CD4^{+}Foxp3^{+}$ cells and (**B**) $CD4^{+}IL17^{+}$ cells in MLN cells were measured by FACS analysis. The data are expressed as the mean ± standard deviation (SD) *p* values of less than 0.05 were considered statistically significant (* *p* <0.05, ** *p* <0.01, *** *p* <0.001). The data are expressed as the mean ± standard deviation (SD).

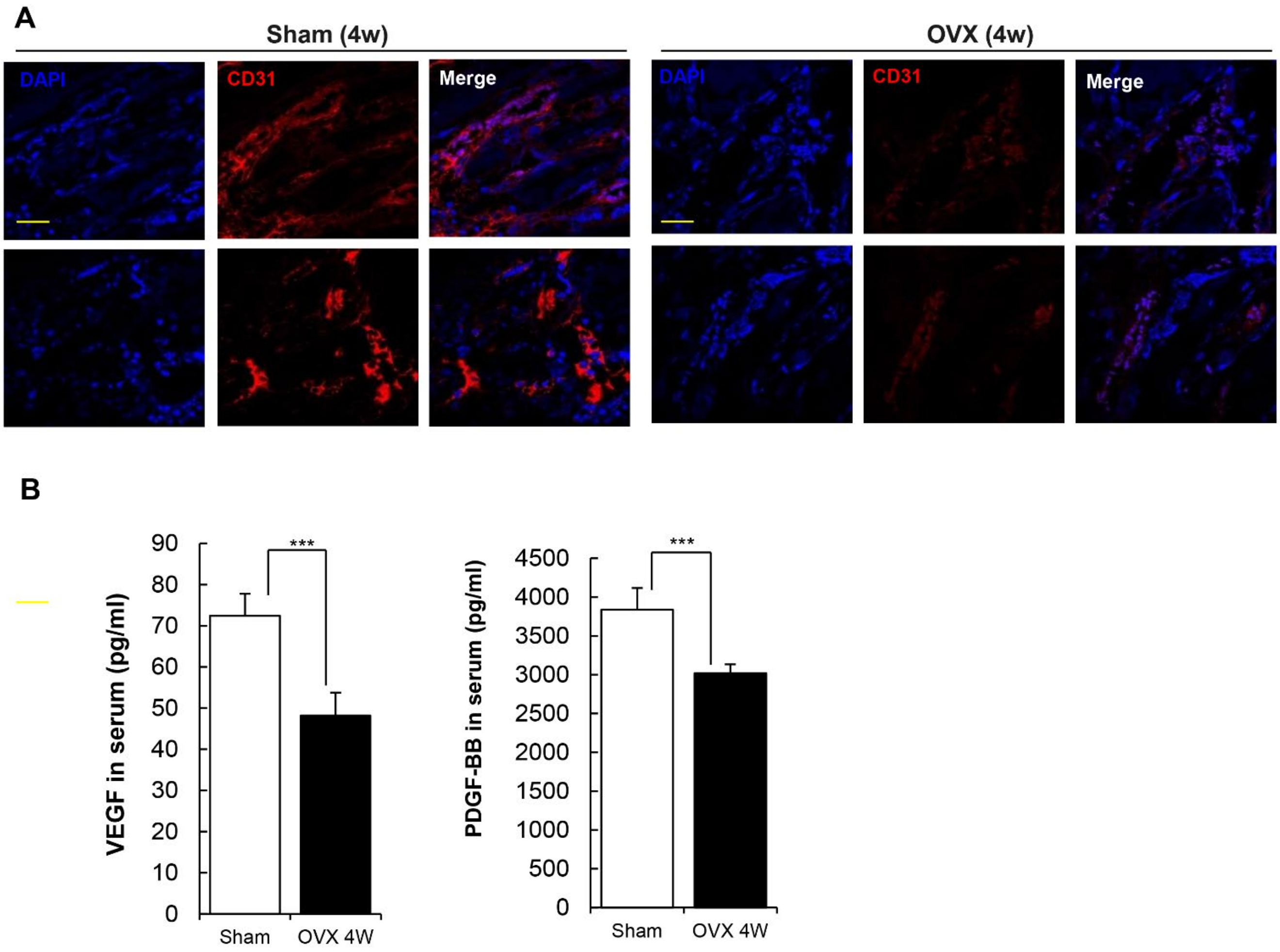

Supplementary Figure 5. OVX induce vessel damages in bone marrow within 4 weeks. (**A**) Representative images of immunofluorescence staining for CD31 (red) in distal femoral metaphysis from rat after sham-operation and OVX for 4 weeks. Scale bar: 200 µm. (**B**) The concentrations of VEGF and PDGF-BB in serum were analyzed by ELISA after 4 weeks of OVX induction and compared with Sham group. *p* values of less than 0.05 were considered statistically significant (* $p < 0.05$, ** $p < 0.01$, *** $p < 0.001$). The data are expressed as the mean ± standard deviation (SD) of three independent experiments.

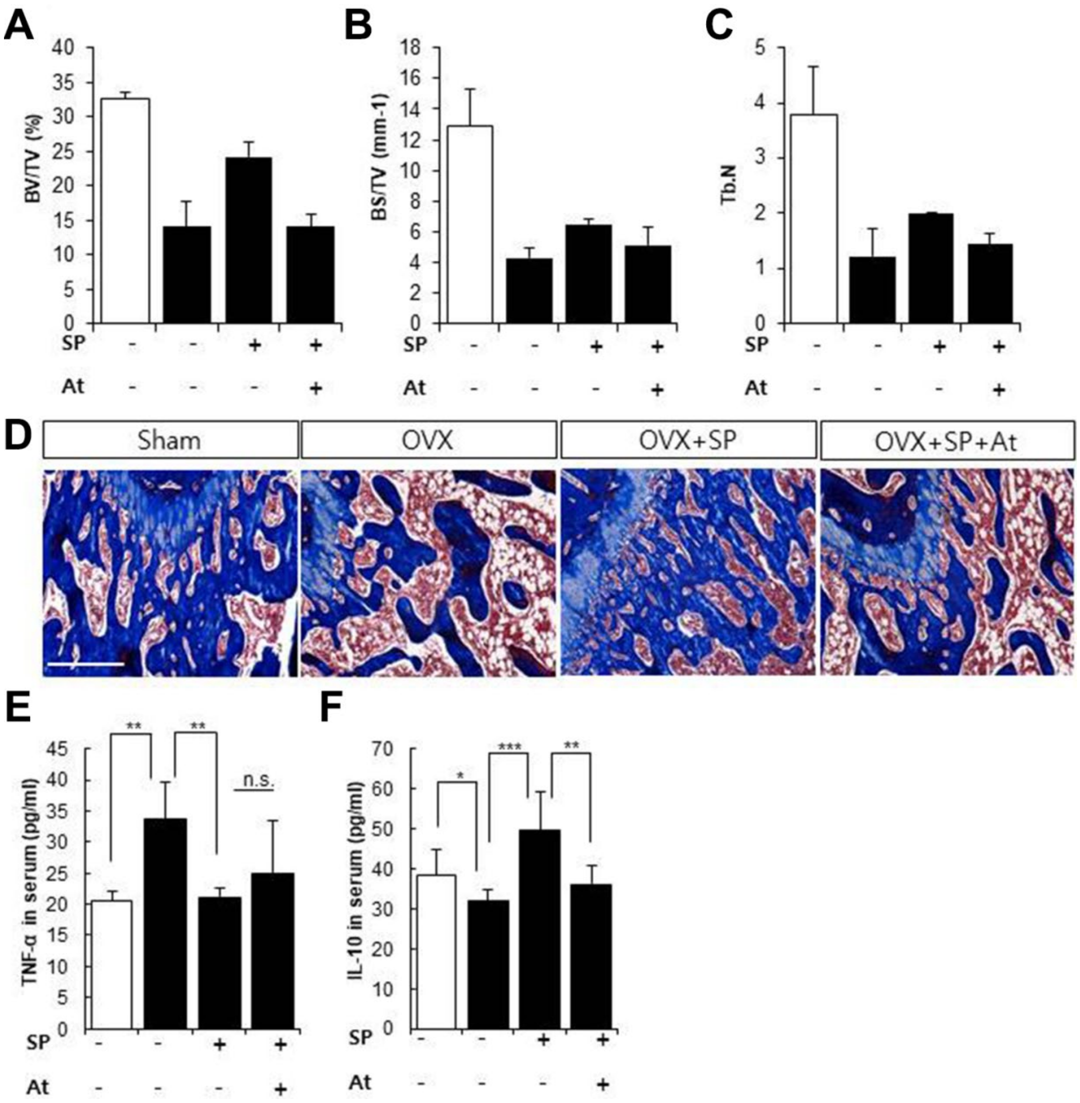

Supplementary Figure 6. SP ameliorates OVX-induced bone loss via binding NK-1R. (**A**–**C**) Quantitative analyses of the trabecular bone fraction of femur from rats. (**D**) Masson's Trichrome staining of distal femoral metaphysis sections from rats after sham-operation and OVX. Scale bar: 200 μm. (**E**, **F**) TNF-α and IL-10 expression levels in serum were analyzed by ELISA. BV: trabecular bone volume. TV: total bone volume. BS: trabecular bone surface. Tb.N: the number of trabecular bone. *p* values of less than 0.05 were considered statistically significant (* $p < 0.05$, ** $p < 0.01$, *** $p < 0.001$). The data are expressed as the mean ± standard deviation (SD) of three independent experiments.

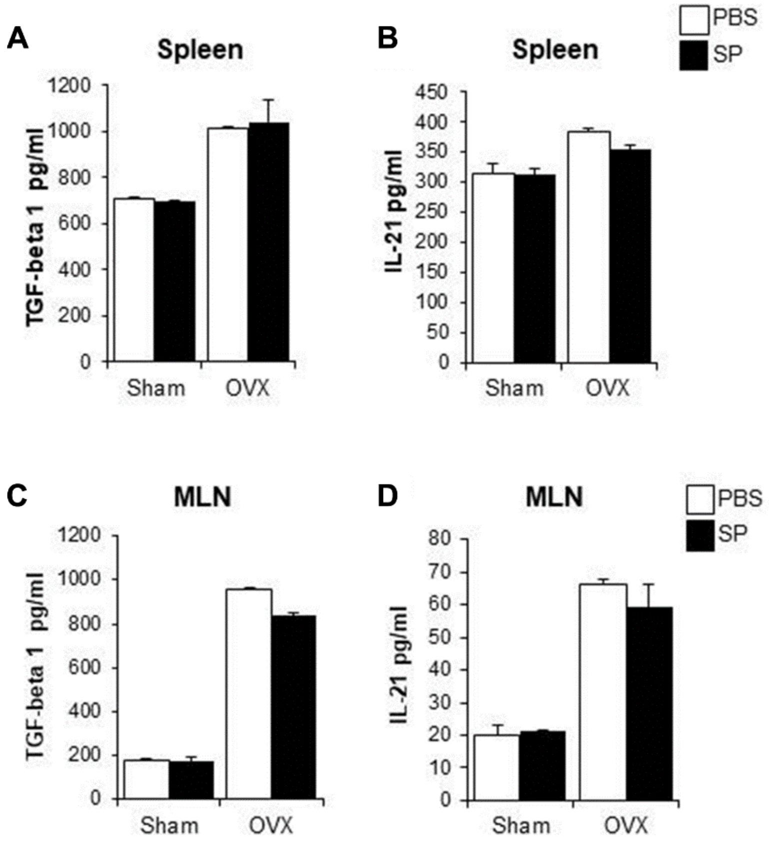

Supplementary Figure 7. SP does not affect TGF-beta and IL-21 production from 2nd lymphoid organ. TGF-beta 1 (**A**) and IL-21 (**B**) expression levels in conditioned medium of splenocytes were analyzed by ELISA. The concentrations of TGF-beta 1 (**C**) and IL-21 (**D**) in conditioned medium of MLN cells were analyzed by ELISA. *p* values of less than 0.05 were considered statistically significant (* *p* <0.05, ** *p* <0.01, *** *p* <0.001). The data are expressed as the mean ± standard deviation (SD) of three independent experiments.

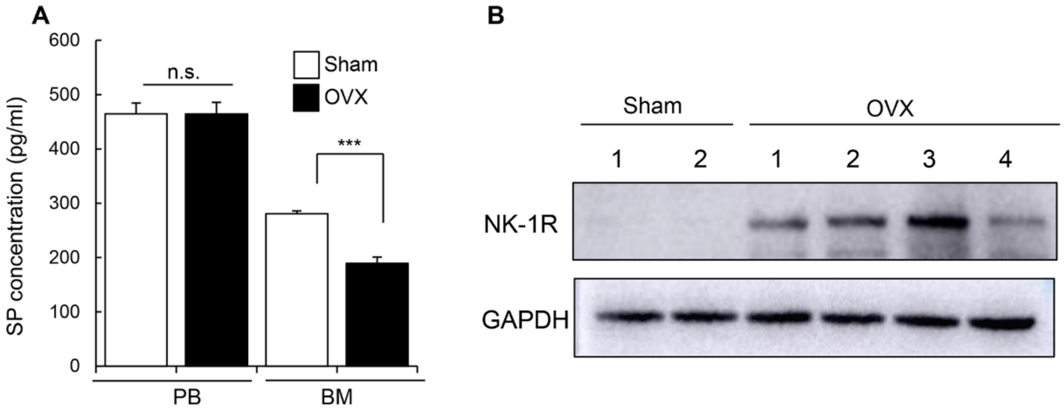

Supplementary Figure 8. OVX downregulates SP levels in the bone marrow but upregulates NK-1R expressions after 8 weeks. (**A**) The concentration of SP in bone marrow aspirates was analyzed by ELISA. (**B**) The level of NK-1R protein in bone marrow was elucidated by western blot analysis and quantified relatively. *p* values of less than 0.05 were considered statistically significant (* *p* <0.05, ** *p* <0.01, *** *p* <0.001). The data are expressed as the mean ± standard deviation (SD) of three independent experiments.
